# Effect of multivitamin and multimineral supplementation on cognitive function in men and women aged 65 years and over: a randomised controlled trial

**DOI:** 10.1186/1475-2891-6-10

**Published:** 2007-05-02

**Authors:** Geraldine McNeill, Alison Avenell, Marion K Campbell, Jonathan A Cook, Philip C Hannaford, Mary M Kilonzo, Anne C Milne, Craig R Ramsay, D Gwyn Seymour, Audrey I Stephen, Luke D Vale

**Affiliations:** 1 Department of Environmental and Occupational Medicine, University of Aberdeen, Foresterhill, Aberdeen AB25 2ZD, UK; 2 Health Services Research Unit, University of Aberdeen, Foresterhill, Aberdeen AB25 2ZD, UK; 3 Department of General Practice and Primary Care, University of Aberdeen, Foresterhill, Aberdeen AB25 2ZD, UK; 4 Health Economics Research Unit, University of Aberdeen, Foresterhill, Aberdeen AB25 2ZD, UK; 5 Department of Medicine for the Elderly, University of Aberdeen, Foresterhill, Aberdeen AB25 2ZD, UK

## Abstract

**Background:**

Observational studies have frequently reported an association between cognitive function and nutrition in later life but randomised trials of B vitamins and antioxidant supplements have mostly found no beneficial effect. We examined the effect of daily supplementation with 11 vitamins and 5 minerals on cognitive function in older adults to assess the possibility that this could help to prevent cognitive decline.

**Methods:**

The study was carried out as part of a randomised double blind placebo controlled trial of micronutrient supplementation based in six primary care health centres in North East Scotland. 910 men and women aged 65 years and over living in the community were recruited and randomised: 456 to active treatment and 454 to placebo. The active treatment consisted of a single tablet containing eleven vitamins and five minerals in amounts ranging from 50–210 % of the UK Reference Nutrient Intake or matching placebo tablet taken daily for 12 months. Digit span forward and verbal fluency tests, which assess immediate memory and executive functioning respectively, were conducted at the start and end of the intervention period. Risk of micronutrient deficiency at baseline was assessed by a simple risk questionnaire.

**Results:**

For digit span forward there was no evidence of an effect of supplements in all participants or in sub-groups defined by age or risk of deficiency. For verbal fluency there was no evidence of a beneficial effect in the whole study population but there was weak evidence for a beneficial effect of supplementation in the two pre-specified subgroups: in those aged 75 years and over (n 290; mean difference between supplemented and placebo groups 2.8 (95% CI -0.6, 6.2) units) and in those at increased risk of micronutrient deficiency assessed by the risk questionnaire (n 260; mean difference between supplemented and placebo groups 2.5 (95% CI -1.0, 6.1) units).

**Conclusion:**

The results provide no evidence for a beneficial effect of daily multivitamin and multimineral supplements on these domains of cognitive function in community-living people over 65 years. However, the possibility of beneficial effects in older people and those at greater risk of nutritional deficiency deserves further attention.

## Background

The suggestion that diet or nutrient supplements could delay or reduce cognitive decline in later life is consistent with the biological effects of B vitamins in lowering homocysteine levels and of antioxidant vitamins and minerals in protecting against tissue damage from reactive oxygen species [1]. Cross-sectional and longitudinal studies have provided some evidence in support of associations between nutrient intake or status and cognitive decline, though the evidence is inconsistent [1]. By contrast, intervention studies with antioxidant vitamins [2-5] or B vitamins [6-8] given for 24 weeks or more in community-living older people have found no evidence for a beneficial effect of supplements, though a recent study of subjects with raised homocysteine levels found a significant benefit of folic acid supplementation for 3 years on global cognitive function and two of the five component cognitive domains [9]. Two randomised trials which used multivitamins for 24 weeks or more also found no evidence of a beneficial effect [10,11] but a small trial of frail elderly people in residential care found a beneficial effect of multivitamin and multimineral supplementation in two of the five tests of cognitive function after six months [12]. Another trial of a multivitamin and multimineral supplement in healthy elderly subjects reported beneficial effects after one year in six of seven tests [13], though these findings have recently been retracted in the light of concerns about the veracity of the data and possible conflicting commercial interest [14].

The MAVIS (Mineral And Vitamin Intervention Study) trial was a large randomised, double-blind, placebo-controlled trial of multivitamin and multimineral supplementation designed to assess possible effects on infection in men and women aged 65 years or over, using a supplement containing eleven vitamins and five minerals [15]. In the MAVIS trial we also collected information on cognitive function to assess the possible effects of multivitamin and multimineral supplementation on cognition, which we present in this paper.

## Methods

The MAVIS trial design and recruitment has been described in more detail elsewhere [15]. 910 men and women aged 65 years and over who had not taken vitamin, mineral or fish oil supplements within three months of recruitment (one month for supplements of water-soluble vitamins other than vitamin B_12_) were recruited from six primary care health centres in North-East Scotland between February and December 2002. 97% of participants in both supplemented and placebo groups were living in the community at recruitment and all were considered by their family doctor to be suitable for the study. We did not exclude any participants on the basis of impaired cognitive function, though those with dementia were unlikely to volunteer or would have been excluded by their doctor. We did not collect information on the educational status of participants but this age group would all have attended formal education for at least seven years.

Participants were randomly allocated to receive either a mineral and vitamin supplement or matching placebo for 12 months, with minimisation on health centre, age, gender and place of residence (living in the community or in care). 456 participants (239 men, 217 women) received the active supplement and 454 participants (240 men, 214 women) received the placebo. The supplement contained 800 μg vitamin A, 60 mg vitamin C, 5 μg vitamin D, 10 mg vitamin E, 1.4 mg thiamin, 1.6 mg riboflavin, 18 mg niacin, 6 mg pantothenic acid, 2 mg pyridoxine, 1 μg vitamin B_12_, 200 μg folic acid, 14 mg iron, 150 μg iodine, 0.75 mg copper, 15 mg zinc, and 1 mg manganese. The participants were asked to take one tablet daily for a 12-month period, with compliance checked by reported consumption at monthly intervals. The nutrient content of the tablet was chosen to be similar to most other commonly used supplements purchased over-the-counter and the dosage was according to the manufacturers' instructions. A 10% random sample provided tablets for tablet counting. Risk of iron, folate, vitamin C or vitamin D deficiency at baseline was assessed using a simple 17 item nutrition risk questionnaire for assessing risk of micronutrient deficiency in older people [16].

Cognitive function was assessed in all participants by digit span forward and verbal fluency tests carried out face-to-face at recruitment and over the telephone at the end of the intervention period. The digit span forward test requires the participant to repeat a sequence of random digits pronounced at a rate of approximately one per second, beginning with two sequences of two digits and continuing with two sequences of increasing length up to a sequence of nine digits or to failures on both tests of a shorter sequence. One point is scored for every correct sequence repeated with a maximum score of 16 points [17]. The verbal fluency test requires the participant to name words beginning with each of three separate letters in an exactly-timed one-minute period. The three letters used (either P, R and W or C, F and L) were randomly allocated at the baseline assessment and the other three letters used at the one year assessment. The score is the total number of words named correctly, excluding proper nouns and words with the same start but different endings [18].

Analyses were conducted with adjustment for baseline values and the trial minimisation factors where appropriate. Intention-to-treat analyses were conducted for trial group comparisons. Statistical significance was based upon the 95% confidence interval. For the two pre-planned trial sub-group analyses (those aged 75 years and over and those at increased risk of deficiency assessed by the nutrition risk questionnaire) the 99% confidence interval was used to give the correct width of the 95% confidence interval.

The study protocol was approved by Grampian Research Ethics Committee and all participants gave written consent to take part.

## Results

The supplemented and placebo groups were well-matched in terms of baseline characteristics (table 1). 290 participants were aged 75 years or over and 260 participants were identified as being at increased nutritional risk at baseline. During the trial, 12 participants died, 77 stopped taking their tablets and 44 were lost to follow up. Compliance with taking the tablets for all participants for the whole 12-month period was over 78% in both supplemented and placebo groups [15].

**Table 1:** Baseline characteristics of the supplemented and placebo groups

Characteristics	Supplemented group (n 456)	Placebo group (n 454)
Age in years: median (interquartile range)	72 (68.0 – 76.0)	71 (68.0 – 76.0)
Body mass index in kg/m^2^: mean (SD)	28.2 (4.2)	27.9 (4.1)
Women: n (%)	217 (48)	214 (47)
Current smoker: n (%)	57 (13)	63 (14)
Past or present hypertension: n (%)	188 (41)	172 (38)
Past or present heart disorders: n (%)	137 (30)	130 (29)
Past or present chest disorders: n (%)	86 (19)	87 (19)
Past or present diabetes: n (%)	37 (8)	42 (9)
Past or present cancer: n (%)	46 (10)	46 (10)
Past or present cerebrovascular disease: n (%)	31 (7)	22 (5)
At risk of iron, folate, vitamin C or vitamin D deficiency: n (%)	145 (32)	117 (26)

There were no significant differences in baseline test results between the supplemented and placebo groups in all participants or in the two pre-planned sub-groups (table 2). Overall there was evidence of improvement in digit span forward test scores from baseline to 12 months (mean change 0.4, 95% CI 0.2, 0.6) but not in the verbal fluency scores (mean change 0.8, 95% CI -0.2, 1.7). There was no evidence for a difference in the overall change between the supplemented and placebo groups in either digit span forward or verbal fluency tests. When separate analyses were repeated for the two subgroups (those aged 75 years or over and those at risk of nutritional deficiency) there was also no evidence for a difference between supplemented and placebo groups in the digit span forward test. However, in the verbal fluency tests in participants aged 75 years or over there was weak evidence for a beneficial effect (mean difference between supplemented and placebo groups of 2.8 (95% CI -0.6, 6.2) units). A similar trend was observed in the participants who were at increased risk of nutritional deficiency (mean difference between supplemented and placebo groups of 2.5 (95% CI -1.0, 6.1) units).

**Table 2:** Cognitive test scores in the supplemented and placebo groups

Participants		Digit span forward scores			Verbal fluency scores		
		
		Baseline Mean (SD)	12 months Mean (SD)	Supplemented vs. placebo Mean diff. (95% CI)	Baseline Mean (SD)	12 months Mean (SD)	Supplemented vs. placebo Mean diff. (95% CI)
All participants	Supplemented	11.0 (2.2) (n 455)	11.5 (2.3) (n 398)	-0.1 (-0.3, 0.2)	32.1 (12.0) (n 454)	33.8 (12.8) (n 397)	0.8 (-0.3, 2.0)
	Placebo	11.2 (2.2) (n 450)	11.7 (2.1) (n 374)		31.7 (12.8) (n 450)	33.0 (13.3) (n 372)	
Participants aged 75 years or over	Supplemented	10.8 (2.2) (n 144)	11.1 (2.3) (n 123)	-0.1 (-0.8, 0.6)^1^	30.0 (12.7) (n 143)	32.4 (13.0) (n 123)	2.8 (-0.6, 6.2)^1^
	Placebo	11.0 (2.1) (n 146)	11.3 (2.2) (n 105)		29.6 (12.5) (n 146)	30.0 (13.4) (n 103)	
Participants at increased risk of deficiency	Supplemented	10.8 (2.1) (n 144)	11.2 (2.4) (n 113)	0.4 (-0.3, 1.1)^1^	29.2 (12.0) (n 144)	31.3 (13.1) (n 113)	2.5 (-1.0, 6.1)^1^
	Placebo	10.8 (2.2) (n 116)	10.9 (2.0) (n 91)		30.6 (13.9) (n 116)	29.2 (13.2) (n 91)	

^1 ^Reflecting the fact that the analysis was carried out on a sub-group, a 99% confidence interval was used to give the correct width for the 95% confidence interval.

## Discussion

Overall, our results provide no evidence for a beneficial effect of multivitamin and multimineral supplementation on cognitive function in the majority of men and women 65 years and over living in the community. This result is consistent with all previous studies in non-selected elderly populations [2-10] apart from the retracted Canadian study [13]. In the present study the doses of the vitamins and minerals in the supplement ranged from 50–210% of the UK reference nutrient intake^a ^i.e. levels which could be achieved by a well-balanced diet. Several other studies have used higher doses, particularly of B vitamins [2-4] or antioxidant vitamins [5-7] but none of these found a beneficial effect. We considered the possibility that the participants in the present study were unusually well nourished but found no evidence to support this, as the proportion of participants aged 75 years and over classified as at increased risk of micronutrient deficiency was similar to those in an earlier cross-sectional survey in the area [19]; in addition we excluded subjects who had been taking dietary supplements prior to the study who were more likely to have had better dietary intake and nutritional status [20].

The cognitive function tests used in the present study assessed only short term memory and executive function, so we cannot rule out the possibility of effects in other cognitive domains such attention and concentration. We selected the two tests used in the present study as they could be carried out by telephone at follow-up, but future studies should include a wider range of tests of different cognitive domains. We also cannot exclude the possibility of effects of nutrients such as selenium or n-3 polyunsaturated fatty acids, which were not included in the supplements used.

For the subgroups defined on the basis of age or nutritional risk, there was weak evidence for a beneficial effect in verbal fluency but not in the digit span forward test. Digit span forward is a test of attention and immediate memory involving a relatively familiar task which may be performed relatively well even in advanced cognitive decline, while verbal fluency tests speed of processing and information retrieval which are sensitive to the difficulty in generating words characteristic of Alzheimer's disease [21]. The possibility that those at increased risk of nutritional deficiency may benefit from supplements is supported by the FACIT study of men and women aged 50–70 y with elevated homocysteine in whom there were significant effects on global cognitive function, though for specific cognitive domains the effects were seen in memory (assessed by recall of 15 common words) and information processing speed (assessed by letter-digit substitution) but not in verbal fluency (assessed by asking the subjects to name as many animals as possible in one minute) or in sensorimotor speed or complex speed [9]. The possibility of a beneficial effect in higher risk subjects is also supported by the study of Dutch care home residents with a BMI less than 25 kg/m^2 ^and median age of 83 y which found beneficial effects of multivitamins and multiminerals in a test of short-term memory and one of two tests of category fluency [12]. The clinical significance of the differences in the verbal fluency test scores between supplemented and placebo groups seen in the present study is difficult to estimate as the tests are not widely used in clinical practice, but data from a cross-sectional study of Canadian adults suggest that the rate of decline in performance on this test is of the order of one point per 3 years [22]. By this estimate the difference of 2 points observed between the supplemented and placebo groups in the present study would be equivalent to the decline seen over 6 years of normal aging and could therefore represent a clinically useful effect.

A limitation of this study is that we classified participants as being at increased risk of nutritional deficiency using a simple risk questionnaire rather than by measuring their actual nutritional status from blood samples. The nutrition assessment questionnaire used had previously been validated by comparison with blood measures of nutrient status in 398 community-living men and women aged 75 and over, in whom the odds ratios (95% CI) for deficiency of iron, folate, vitamin C and vitamin D in those identified at risk by the questionnaire were 3.8 (2.0, 6.9); 5.1 (2.3; 11.3); 5.7 (3.1, 10.4) and 4.3 (1.9, 9.4) respectively [16]. Better definition of subjects at nutritional risk could be useful in assessing whether the effect seen in those identified as being at nutritional risk in this study is a real effect.

In summary, we found no evidence for a beneficial effect of multivitamin and multimineral supplementation on two tests of cognitive function in tests in the total sample of participants, but cannot exclude the possibility of beneficial effects in those at higher risk of nutritional deficiency. In view of the high social and economic costs of cognitive decline and the low cost of nutritional supplementation, further intervention studies in older people, particularly those at increased risk of nutritional deficiency, are still warranted.

## Note

^a^Note: in the UK there are no RDAs for vitamin E, pyridoxine, pantothenic acid and manganese
